# Patience with foster children as a major problem for grandparents in the role of kinship carers aged 60 + and the psychosocial determinants of this deficit

**DOI:** 10.1186/s12877-023-04512-x

**Published:** 2023-12-05

**Authors:** Marta Giezek, Anna Wojtkowska, Paulina Zabielska, Beata Karakiewicz

**Affiliations:** 1 Subdepartment of Social Medicine and Public Health, Department of Social Medicine, Pomeranian Medical University in Szczecin, Szczecin, 71-210 Poland; 2 Faculty of Psychology in Wroclaw, SWPS University, Warsaw, Poland

**Keywords:** Patience, Foster care, Grandparents, Stress coping, Sense of coherence

## Abstract

Kinship care represents the most prevalent form of foster care in Poland. Most commonly, the role of kinship carers is taken on by grandparents, who may struggle with various problems, needs and deficits in this role. The aim of this study was to investigate the problem of patience in kinship carers aged 60 + and its impact on deficits in the performance of roles and duties.

**Methods** Seventy-five foster grandparents (63 female, 84%) aged from 61 to 97 years (M = 69,12; SD = 6.22) were investigated in north-western Poland in 2018 and 2019. The study was based on the diagnostic survey method.

**Results** Psychological disposition, functioning, health problems and parental needs and deficits were assessed using standardised psychometric scales and tools self-constructed for this research study. A lack of patience with foster children was reported by 46.7% (*n* = 35) of the respondents. Patience deficits corresponded with a significantly lower sense of coherence, especially in the manageability domain (*p* < 0.001) and such stress coping strategies as lower positive reappraisal (*p* = 0.016) and seeking of emotional support (*p* = 0.025), as well as a greater tendency for suppression of activities (*p* = 0.014) and venting of emotions (*p* = 0.035). Relatively permanent personality traits and general self-efficacy were not differentiated by patience with children.

**Conclusions** The results suggest that patience – so important for biological and foster parents – is related to psychological competencies that can be improved through psychoeducation and skills-training, which may be beneficial for improving foster carers’ effectiveness.

## Background

The Oxford Dictionary defines patience as “the ability to stay calm and accept a delay or something annoying without complaining” [1]. Being a parent is a great gift and a privilege, but also a responsibility, as children’s life chances depend heavily on the resources of the family into which they are born [2]. Parenthood powerfully affects the human capacity for patience. Bauer and Chytilova (2013) found that among childless partners the difference in patience between men and women was minimal, but it increased to a statistically significant level for partners with small children [3]. Parental patience is put to the test time and again. There is no doubt that the ability to endure difficult and even distressing situations with calm and composure is essential in a relationship with a child. A child or young person is constantly learning and experimenting, exploring the world, themselves and other people. They are not born knowing what is right or wrong, they make mistakes, forget and test boundaries. Some of their behaviour will be appropriate, but some will be undesirable. It is the parent’s job to help the child distinguish between the two. And, of course, to keep answering the „why” questions. In such situations, being patient, explaining and refraining from impulsive reactions will be parental allies [4]. The attainment of patience is a difficult multi-stage task which requires long-term effort and is requisite at every stage of life as well as in family, professional and social activities. Therefore, older family members are of particular importance in intergenerational relationships. Thanks to their life experience and patience built over many years, they constitute a value in the family, and in extreme situations, when parents lose or have limited child custody, it is grandparents who most frequently take over this responsible role [5].

Kinship foster care means that the functions and roles of biological parents are taken over by other family members. From a systemic perspective, the family is a system with a certain set of norms and rules, as well as measures to sustain its cohesion. The family system has specific goals, as well as ways of meeting the needs of individual family members and conducting basic social duties. This system has its own homeostatic capabilities, i.e. striving for a specific type of internal balance, which is essential to prevent family disintegration [6]. The family is constantly trying to overcome crises resulting from changes in its members, being joined by new members, work-related stress, health problems, dysfunctions, violence, addictions or special life circumstances. Therefore, the process of family continuation is characterized by unstoppable dynamic changes [7]. However, thanks to the self-stabilizing capabilities of the family system, it has a tendency to survive.It is worth the effort, as more patient parents will help their children learn how to cope with everyday problems in a calm way by observing how to be patient in their own lives. In a very recent longitudinal study, children’s patience significantly predicts further academic track choices, and this link stays robust even after controlling for family background, school-class fixed effects, risk preferences, and cognitive abilities [8]. At the same time, impatience is a strong predictor of criminality among young men [9]. Patience improves stress, tolerance and coping, both in daily situations (like studying: [10]) and in extreme circumstances (like the COVID-19 pandemic: [11]). As a coping strategy for interpersonal stressors, it is associated with lower depression symptoms [12]. Among mothers raising children with autism spectrum disorder, remaining patient helps to restrain feelings of anxiety, despair and anger, which leads to more adaptative coping strategies [13, 14]. Authoritative parents (adequately controlling and warm at the same time) are more likely to encourage patience in children [15], but many studies show that this ability can be raised through skills training and psychoeducation [16]. For every child, anywhere in the world, the family should be the best environment to live and grow, but not all parents are able to exercise their parental responsibility as required by the best interests of the child. Some of them are able to benefit from support in the form of social work, i.e. social services [17] aimed at restoring their ability to perform parental roles. Support of such parents involves the analysis of the family’s situation and the reasons for the existing crisis, raising awareness as to family planning and functioning, improving parenting skills and assistance with respect to the process of family reintegration. Some parents, however, despite the commitment of professionals and use of all possible forms of assistance, continue to fail in their role as parents, as a result of which the child’s development, safety, health and even life may be put at risk [18]. In such families, children experience neglect, trauma and abuse [19]. In such situations, the child may be placed in alternative care, which is aimed at providing temporary care and support, by offering stability, continuity and safety of a foster home, as well as therapeutic interventions to help the child overcome their problems, as detailed in Table 1.

**Table 1:** Structure of foster care in Poland as of 31 December 2018

Type of foster care	Number of providers	Number of children
Total	38,377	72,339
Institutional foster care	1,125	17,051
Family foster care	37,252	55,288
- Including: kinship care	23,544	30,185

Source: Publication of the Ministry of Family, Labour and Social Policy based on material and financial reports on the performance of tasks related to family support and foster care system (status as of 31 December 2018)

Because of the fact that the family is the best and natural source of support for the child, prospective foster parents are in the first place sought among the child’s relatives. The study by M. Oosterman et al. confirmed that a positive relationship with foster carers affects children’s ability to regulate emotions [20]. Kinship care represents the most prevalent form of foster care in Poland. In this model, care is provided by the child’s siblings or ascendants – the grandparents. In the latter case, an elderly person must again assume the very demanding and responsible role of a parent, caregiver. Furthermore, older people struggle with various age-related problems and social consequences of the ageing process, including notably: changes in health and psychosocial status, withdrawal from the labour market and lower economic status [21, 22]. Placing a child in kinship care is intended to protect the child from threats that may exist in the biological family, and to provide an opportunity to restore peace of mind and harmonious development in a healthy environment. Most of the problems experienced by foster carers in their day-to-day functioning are directly related to the reasons why children were placed in a foster family. The main reasons for placing children in foster care are due to the dysfunctional nature of the biological family – parental addiction (41.7%), helplessness in care and upbringing (28.1%), domestic violence (3.8%). On the other hand, 4% of children placed in foster care are orphaned, 7.3% are semi-orphaned, and 2.2% of children are so-called “Euro-orphans”, where at least one parent has emigrated to work abroad (https://www.nik.gov.pl/aktualnosci/nik-o-opiece-na-dziecmi-w-pogotowiach-opiekunczych-i-rodzinnych.html). It is usually difficult to identify a clear dominant cause, as it tends to be a combination of unfavourable conditions, the coexistence of many structural and functional problems of these families and the related crisis situations. The child comes with an emotional baggage of experiences and traumas that are often known to the grandparents, who face a difficult task when taking on foster care, perhaps combined with a sense of their own inadequacy as parents [23]. The subject of patience is not popular in research, especially in such a narrow field as kinship care. There is, however, a noteworthy analysis of patience written by T. Borys, who demonstrates that patience has its origin in the emotional sphere and is a basic form of self-defence against impulses that arouse various fears, anxieties and growing impatience with threats that, according to popular opinion, last too long.. Borys’s research on patience is of a universal nature, but in the context of foster care provided by grandparents, it is relevant to consider his description of people living in a civilisation of impatience when confronted with threats [24]. The rush of everyday life mentioned above, differences between generations and responsibilities do not encourage the patience that foster carers should have in abundance. Our practical observations revealed that many foster grandparents report deficits of different psychosocial skills important in caregiving. In addition to material needs and insufficient knowledge of child and adolescent development, many carers report a lack of patience with growing foster children. There is very little research on the role of patience in the family system and none involving foster families. Based on the available literature, it was found that our current knowledge of the determinants of patience and methods of enhancing it is limited. At the same time, practical experiences in social work with foster parents suggest that certain dispositions and skills coexist with a better ability to remain patient even in difficult situations. Thus, the aim of this study was to analyse the role of impatience among the various problems reported by foster families and its relationship with certain psychological dispositions in order to gain a better understanding of this phenomenon.

To achieve this aim, we analysed kinship families in north-western Poland, where the carers were aged 60 years and older and cared for their grandchildren. Based on the analysis of documentation, 189 such families were identified.

According to Table 2, 63 people provide kinship care on their own, without the support of a spouse or partner. Grandparents as foster carers, even though officially appointed by the court, are not professionals. In their role, sanctioned by law, they rely largely on previous life experience and family relations, which may be compared to the concept of an informal carer, that is a person who provides help and support free of charge to a partner, child, relative, friend or neighbour, who otherwise would not be able to cope without such help [25]. The key objective of this study was to investigate the problem of patience in kinship carers aged 60 + and its impact on deficits in the performance of roles and duties. Two research assumptions were tested in this study:

1. A lack of patience with foster children is a frequent deficit in kinship care reported by elderly foster carers and is not different between genders.
2. A lack of patience differentiates personality traits, sense of coherence, general self-efficacy and stress coping strategies.

**Table 2:** Structure of kinship families with carers aged 60 years and older in north-western Poland

Number of kinship families with carers aged 60 +	Number of children aged 0–6	Number of children aged 7–15	Number of children aged 15–18
189 families 252 carers	23	127	82

## Methods

The manuscript is based on a portion of research carried out within the framework of the research project: “Identification of problems in the medical, psychological and social sphere in people aged 60 years and older who – pursuant to a court decision – provide kinship care to their grandchildren”. The study was conducted in 2018–2019 in north-western Poland. The target population comprised 189 families with kinship carers over the age of 60 years. Consent to participate was obtained from 75 carers, that is 39.7% of those eligible for the study. Each participant was given information about the aim and procedure of the study with a written assurance that they can withdraw at any stage of the research process, without having to provide information as to the reasons for the decision.

The study was based on the diagnostic survey method. Lack in patience was measured subjectively by foster carers in response to a direct question in the survey.

The question was “What do you find most lacking in your role as a kinship carer?”. One of the answers was “patience”. The respondent could answer yes or no. The deficits listed in this part of the survey were selected by the authors on the basis of the experiences of foster care professionals.

Given the lack of research in this area, and inspired by our own practical experience of social work with foster families, we decided to test an initial hypothesis about the relationship between parental patience and four inventories of psychological dispositions:

1. GSES (General Self-Efficacy Scale), which is used to assess the general belief in one’s competence to cope effectively with difficult situations and obstacles. It consists of 10 items assessed on a scale from 1 (“not at all true”) to 4 (“exactly true”). The scale has been adapted to 21 populations worldwide and its validity has been confirmed in Poland, showing very good reliability with Cronbach’s alpha higher than 0.85 (Juczyński, 2001) [26].
2. SOC-29 (Antonovsky’s Life Orientation Questionnaire), which explores the sense of coherence, comprised of the ability to understand events (comprehensibility), to cope (manageability) and to find meaning (meaningfulness) in commitment and creating one’s life. This tool consists of 29 items assessed on a Likert’s scale from 1 to 7. In the Polish population, it has a proven validity and acceptable reliability, achieving Cronbach’s alpha from 0.68 to 0.78 for individual subscales and 0.92 for the global score (Koniarek, Dudek, Makowska, 1993) [27].
3. Mini-COPE, which is a measure of dispositional coping, i.e. typical responses and feelings associated with severe stress (including active coping, planning, seeking support, acceptance, positive reframing, humour, religion, psychoactive substance use, denial, disengagement, etc.). It consists of 28 items assessing 14 coping strategies (two items for each), scoring on a scale from 0 (“I almost never do this”) to 3 (“I almost always do this”). As it is very short, with two items for each strategy, this tool has a satisfactory reliability in the Polish population (Cronbach’s alpha from 0.62 to 0.89) and proven validity (Juczyński, Ogińska-Bulik, 2009) [28].
4. NEO-FFI (NEO Five Factor Inventory), which examines personality traits included in the popular Big Five model: neuroticism, extraversion, openness to experience, agreeableness and conscientiousness. This questionnaire consists of 60 items assessed on a scale from 1 (“totally disagree”) to 5 (“totally agree”) and has a confirmed validity and excellent reliability (Cronbach’s alpha from 0.68 to 0.82) (Zawadzki et. al. 1998) [29].

Medical problems were identified by analysing the individual primary care nursing care sheets, in the form applicable in Poland, as provided for in Annex No. 8 to the order of the President of the National Health Fund No. 69/2007/DSOZ of 25 September 2007. The sheet contains the subject’s personal details, as well as information about their health, physical and mental condition, members of the household and living conditions.

To determine the profile of the kinship carer aged 60 years and older, including: social background, education, occupational status, material status, marital status, having own biological children, concerns and problems arising from the role of foster parent, we used a survey of our own design based on the experiences of professionals dealing with issues in foster care.

The surveys were administered by a trained member of staff at the kinship family home. The study design obtained the approval of the Bioethics Committee KB-0012/166/03/18.

### Statistical analysis

Statistical analysis was performed using the IBM SPSS Statistics package v. 25. The sociodemographic characteristics of the group included the analysis of the number of observations (N and %) for quantitative variables, as well as means (M) and standard deviation (SD) for numeric variables. The differences between men and women were analysed using non-parametric tests: Pearson’s chi-squared test and Mann–Whitney U test. In the analysis of differences in selected psychological attributes between individuals reporting and those not reporting lack of patience with foster children, the descriptive statistics for the entire sample included M and SD of test results, followed by the median (Me) and interquartile range (IQR) for groups, which were subsequently compared using the non-parametric Mann–Whitney U test. The statistical significance level was set at *p* < 0.05.

### The group

In total, 78 carers participated in the study, but the results of 3 people were excluded from the analysis due to missing data in at least one of independent variables. In the end, the analysed sample comprised 75 kinship carers (with 84% women), aged between 61 and 97 years. The detailed characteristics of the study participants are presented in Table 3. For numeric variables (age, living space, duration of foster care), we provided means (M) and standard deviation (SD), while the frequency (N) and percentage (%) described the rest of quantitative variables. In the majority of sociodemographic characteristics, no differences were observed between the studied men and women – the only significant difference was found in marital status. While the majority of the studied men were married, among the women more than 40% were widows. Moreover, the divorce rate among women was more than double that for men.

**Table 3:** Sociodemographic characteristics of the sample, including gender differences

Variables	Whole sample, *N* = 75	Women, *n* = 63	Men, *n* = 12	Gender comparison		
	M(SD) / N(%)	M(SD) / N(%)	M(SD) / N(%)	X2 / Z	df	p
Age	69.12 (6.27)	68.85 (6.15)	70.50 (6.96)	-0.955	-	0.340
Education						
Primary	19 (25.3%)	17 (27.0%)	2 (16.7%)	5.380	4	0.250
Vocational	28 (37.3%)	21 (33.3%)	7 (58.3%)			
Secondary technical	9 (12.0%)	8 (12.7%)	1 (8.3%)			
Secondary comprehensive	12 (16.0%)	12 (19.0%)	0 (0.0%)			
Tertiary	7 (9.3%)	5 (7.9%)	2 (16.7%)			
Social background						
Middle class	11 (14.7%)	10 (15.9%)	1 (8.3%)	1.129	2	0.569
Working class	52 (69.3%)	44 (69.8%)	8 (66.7%)			
Farming	12 (16.0%)	9 (14.3%)	3 (25.0%)			
Marital status						
Married	37 (49.3%)	26 (41.3%)	11 (91.7%)	10.723	2	0.005
Divorced	11 (14.7%)	10 (15.9%)	1 (8.3%)			
Widowed	27 (36.0%)	27 (42.9%)	0 (0.0%)			
Living space (in sqm)	59.89 (27.19)	57.00 (19.63)	74.58 (49.53)	-1.295	-	0.195
Duration of foster care	9.61 (6.03)	10.06 (6.27)	7.33 (4.07)	-1.387	-	0.165
Number of children in foster care						
One	48 (64.0%)	40 (63.5%)	8 (66.7%)	1.587	3	0.662
Two	20 (26.7%)	16 (25.4%)	4 (33.3%)			
Three	4 (5.3%)	4 (6.3%)	0 (0.0%)			
Four and more	3 (4.0%)	3 (4.8%)	0 (0.0%)			
Self-assessment of housing conditions						
Very bad	0 (0.0%)	0 (0.0%)	0 (0.0%)	0.960	2	0.619
Bad	0 (0.0%)	0 (0.0%)	0 (0.0%)			
Average	17 (22.7%)	13 (20.6%)	4 (33.3%)			
Good	38 (50.7%)	33 (52.4%)	5 (41.7%)			
Very good	20 (26.7%)	17 (27.0%)	3 (25.0%)			
Self-assessment of financial situation						
Very bad	0 (0.0%)	0 (0.0%)	0 (0.0%)	3.390	3	0.335
Bad	2 (2.7%)	1 (1.6%)	1 (8.3%)			
Average	37 (49.3%)	32 (50.8%)	5 (41.7%)			
Good	30 (40.0%)	26 (41.3%)	4 (33.3%)			
Very good	6 (8.0%)	4 (6.3%)	2 (16.7%)			

## Results

### Deficits reported by carers

Table 4 contains data on the frequency of reported deficits. The respondents most often mentioned lack of patience with their foster children – which was reported by nearly half of all the respondents, including 40% of the women and close to 60% of the men. The second most-prevalent problem experienced by the carers was the need for better housing conditions (nearly 30% of the group), and the third – shortage of money, reported by almost one in every four respondents. The least frequently reported problems (< 10%) included lack of knowledge about children’s needs and development, preventing risks and communicating with an adolescent, as well as lack of institutional support. Gender was not a significant differentiating factor with respect to the deficits reported by kinship carers.

**Table 4:** Frequency of reported deficits in kinship care, including gender differences

Deficits reported by carers	Whole sample, *N* = 75	Women, *n* = 63	Men, *n* = 12	Gender comparison		
	N (%)	N (%)	N (%)	X2	df	p
Patience	35 (46.7)	28 (44.4)	7 (58.3)	0.781	1	0.530
Better housing conditions	22 (29.7)	19 (30.6)	3 (25.0)	0.153	1	0.999
Money	17 (23.6)	16 (26.2)	1 (9.1)	1.518	1	0.440
Family support	13 (17.6)	12 (19.4)	1 (8.3)	0.843	1	0.679
Knowledge on communicating with an adolescent	7 (9.7)	6 (9.8)	1 (9.1)	0.006	1	0.999
Knowledge on risk prevention	6 (8.1)	4 (6.5)	2 (16.7)	1.408	1	0.249
Institutional support	6 (8.1)	5 (8.1)	1 (8.3)	0.001	1	1.000
Knowledge on children’s needs and development	2 (2.7)	1 (1.6)	1 (8.3)	1.767	1	0.296

Lack of patience can be attributed to a number of factors, including cultural background, upbringing, education, and technological advances that accelerate the pace of life [22]. However, it cannot be excluded that lack of patience is also related to living and financial conditions. In view of the fact that the surveyed group of kinship carers aged 60 + mentioned the need for better living and financial conditions as the second or third most important problem after lack of patience, it is possible that improved living conditions in this respect would have a positive effect on patience.

### Psychosocial correlates of the lack of patience in carers

Table 5 shows the analysis of the differences in chosen psychological dispositions depending on the patience status reported by the caregivers. The first column contains the minimum and maximum values, mean and standard deviation for the study sample as a whole. The next two columns contain the median and interquartile range for the compared subgroups, and the results of median-based Mann–Whitney U test. The data were limited to significantly differentiated variables only. There were no statistically significant differences (*p* > 0.05) in terms of the relatively permanent personality traits (NEO_FFI) and general self-efficacy (GSES).

**Table 5:** Comparison of psychometric scores according to carer’s patience with foster child

Variables	Scales	Whole group; min–max. M(SD)	Lack of patience with foster child; Me (IQR)		Mann–Whitney U test	
		*N* = 75	NO, *n* = 40	YES, *n* = 35	Z	p
Coherence	Comprehensibility	23.00—77.00 56.09 (12.03)	61.50 (13.25)	53.00 (15.00)	-2.519 *	0.012
	Meaningfulness	17.00—56.00 46.27 (8.02)	49.00 (6.00)	46.00 (10.00)	-2.278*	0.023
	Manageability	57.00 (16.00)	61.50 (13.25)	53.00 (15.00)	-4.366*	< 0.001
Stress-coping strategies	Active coping	0.00—6.00 4.76 (1.26)	5.00 (2.00)	5.00 (2.00)	-1.418	0.156
	Planning	1.00—6.00 4.59 (1.24)	5.00 (2.00)	5.00 (1.50)	-0.108	0.914
	Positive reappraisal	1.00—6.00 4.07 (1.31)	4.50 (1.00)	4.00 (1.00)	-2.418*	0.016
	Acceptance	2.00—6.00 4.54 (1.19)	4.50 (1.00)	4.00 (2.00)	-0.424	0.672
	Sense of humour	0.00—5.00 0.89 (1.19)	1.00 (2.00)	0.00 (1.00)	-0.745	0.457
	Turning to religion	0.00—6.00 2.79 (2.35)	3.00 (4.25)	4.00 (5.50)	-0.071	0.944
	Seeking emotional support	0.00—6.00 3.66 (1.67)	4.00 (2.00)	3.00 (2.00)	-2.238*	0.025
	Seeking instrumental support	0.00—6.00 3.04 (1.79)	4.00 (2.00)	3.00 (3.00)	-1.471	0.141
	Dealing with something else	0.00—6.00 3.28 (1.64)	3.00 (2.00)	4.00 (3.00)	-1.816	0.069
	Denial	0.00—6.00 1.59 (1.39)	1.00 (2.00)	2.00 (2.00)	-1.531	0.126
	Venting of emotions	0.00—6.00 1.87 (1.52)	1.00 (2.00)	2.00 (2.00)	-2.106*	0.035
	Use of psychoactive substances	0.00—6.00 0.15 (0.79)	0.00 (0.00)	0.00 (0.00)	-0.144	0.885
	Suppression of activities	0.00—6.00 1.17 (1.45)	0.00 (1.25)	2.00 (2.50)	-2.456*	0.014
	Self-blame	0.00—6.00 2.45 (1.72)	2.00 (3.00)	3.00 (3.00)	-1.081	0.280

^*^p < 0.05

Impatience with foster children significantly differentiated all the indicators of the sense of coherence. The greatest differences were observed in manageability (*p* < 0.001). The median score in this scale for people who reported lack of patience with children was as much as 11 points lower than that of their more patient counterparts. Significantly lower median scores were also noted in comprehensibility (by 8.5 points) and meaningfulness (3 points).

Out of the 14 stress-coping strategies, lack of patience was a significant differentiating factor for four strategies (*p* < 0.05). One more represented a non-significant trend.

The first coping strategy where significant differences were observed was the positive reframing technique. Higher scores among people who did not report lack of patience with children are indicative of a greater capacity to see the positive side and learn from difficult situations. People who had no problems with patience also obtained higher scores in the functional strategy of seeking emotional support. In this strategy, stressful situations prompt the person to seek an outlet for their emotions, which helps regulate tension and facilitates the coping process. Two further strategies are less adaptive and were more pronounced in carers who declared lack of patience with children. These were venting and behavioural disengagement. The former is more active, but does not in any way improve the problematic situation. While venting helps regulate emotions, it does so in an unconstructive way, often hurtful to others. Behavioural disengagement, on the other hand, is a passive, avoidance-based behaviour – when relying on this strategy, we get no closer to solving the problem, nor to regaining emotional equilibrium, while chronic avoidance coping over the long term only makes matters worse as well as generating additional problems. Besides the higher rates of behavioural disengagement in people reporting lack of patience, we also observed a tendency to adopt another avoidance strategy – self-distraction. Escaping into alternative activities may have similar effects as disengaging.

Apparently, the psychosocial determinants of the lack of patience with children in kinship care are based on personal competencies – characteristics which can be intentionally strengthened or weakened through psychoeducation or therapy. Attributes such as the sense of coherence or use of more constructive stress-coping strategies may change over time and under the influence of external and internal factors. Interestingly, relatively permanent personality traits did not differ depending on patience with children, suggesting even more strongly that the characteristics which determine lack of patience are malleable.

## Discussion

The features attributed to old people express some ambivalence. On the one hand, old age is characterized by life wisdom, experience, rational thinking and views, responsibility, constructive reflection and self-control. On the other hand, it brings to mind terms such as sadness, discontent, grumpiness, confusion, lack of patience, as well as excessive, unrealistic expectations and taking things for granted [30]. The negative perception of old age from the angle of the decreasing physical abilities of older people and changes in their cognitive processes leads to stereotypes which frequently present a prejudiced image of old people [31]. A positive approach to the issue acknowledges that old age and youth are two different stages of life which perfectly complement each other and provide generations with opportunities to learn from each other [32].

Europe-wide, the percentage of grandparents caring for grandchildren falls in the range of 20–40%, with higher levels observed in southern countries, and lower in Scandinavia [33]. In Australia, 16,000 grandparents take on the role of primary carers for their grandchildren. In the United States overall 3.6% of grandparents live with their grandchildren, and 42% of them provide for the majority of the basic needs of grandchildren under 18 [34]. In such situations, the roles and obligations of grandparents become the roles and obligations of parents, and all entitlements arise from the commitments they take on.

The academic literature contains a large body of publications on professional and non-relative foster care, while very little information can be found in published research about kinship care arrangements involving grandparents and grandchildren with reference to the essential competency of patience, hence the problem with elaborating discussion and comparing research findings. The study by Z. Dalir, A. Heydari, H. Kareshki, Z. S. Manzari [35] reveals that patience is important for creating a peaceful environment for the child, which in turn has positive effects on the physical and psychological health of family members. The participants of that study practised tolerance for the difficult conditions of caregiving, controlled themselves and behaved patiently. According to the authors, these actions resulted from the sense of commitment and responsibility for the care of not only the child, but also other family members to maintain a calm atmosphere in the family. These observations are consistent with the findings of our study. Grandparents in the role of foster parents who had no problems with patience were more capable of seeing the positive side and learning from difficult situations. They were also more likely to seek emotional support for coping with care-related problems. These findings correspond with other research studies explaining how patience can buffer negative tension and lead to more constructive coping, better adaptation to living conditions and maintenance of well-being [36]. Besides coping strategies, this research provides new and original evidence about the strong connection between patience and the sense of coherence, which is also a determinant of successful management in different life events. The sense of coherence helps people see themselves as able to deal with unpredictable and unexpected circumstances. In particular, manageability was found to counteract impatience in foster grandparents, which may be crucial in terms of potential for improving patience. Our results show that the phenomenon under study is determined by fully changeable dispositions, contrary to stable personality traits and general self-efficacy. This appears to provide a positive starting point for further research into how we can train parents and foster parents to be more patient by taking advantage of constructive coping strategies and a greater sense of coherence. As suggested by other studies, the ability to remain patient even during stressful events will not only improve general control, effectiveness and satisfaction, but can also be strengthened via skills-trainings and psychoeducation [20, 36–38].

The strength of our study is that its findings can be applied in practice to develop educational, supporting and psychological interventions, prepared by professionals for grandparents providing kinship care to their grandchildren, so that by learning to be patient they can cope more effectively with caregiver stress and responsibility.

## Conclusions

The psychosocial determinants of the lack of patience with foster children in kinship care are related to personal competencies – characteristics which can be intentionally strengthened or weakened through psychoeducation or therapy. Relatively permanent personality traits were not differentiated by patience with children, suggesting that the characteristics which affect lack of patience are malleable.

The process of increasing the level of patience in grandparents is also dependent on their health, housing conditions and financial situation. Providing kinship foster families with not only psychological support, but also social and living assistance will certainly have a positive effect on their sense of well-being and, consequently, their patience. Older people who take on the responsible role of foster parents to their grandchildren, due to their age, often struggle with multi-morbidities as well as physical and cognitive limitations. A form of institutional support in this area might be financing respite care, thanks to which grandparents will be able to take better care of themselves, which will increase their level of patience.

The patience of grandparents providing kinship care to their grandchildren reflects a conscious commitment to play an active role in their lives, a hope to improve their fate and a willingness to take certain actions for their well-being. Patience can also be a source of strength, making it easier to survive crises, difficulties, disorders, inhibitions or limitations and mistakes. Therefore, if there are any factors that may help improve the patience of grandparents as kinship carers towards their grandchildren, this would be a valuable contribution to the direction of efforts by foster care support services. Is it possible to provide foster care without patience? This question remains open, and requires a separate in-depth analysis addressing not only the relevance of patience in this process, but also the factors that will help strengthen it. When analysing forms of assistance and support for kinship care in everyday functioning and in difficult situations, it will be useful to provide for the improvement of grandparents’ skills as foster carers in dealing with their grandchildren’s problems, e.g. in dealing with trauma, aggression, low self-esteem, rejection and in recognising their own strengths and weaknesses. Carers in the 60 + age group need support to cope with changing realities and technological advances, e.g. in terms of computer literacy and tackling online abuse (cyberbullying) and addiction. Support measures also need to take into account financial and living conditions, the satisfaction of which can have an impact on patience.

Therefore, support for grandparents as foster carers must be comprehensive and multidimensional, with equal importance being attached to psychoeducation, therapy, provision of social, living and financial comfort as well as the ability to take care of their own needs, and not only the needs of their grandchildren.

## Limitations

Obtaining consent for participation was a serious obstacle in the research process. Out of the 189 families meeting the eligibility criteria, only 75 families gave their consent. It should not be ignored that children are placed in kinship care by the family court, and kinship carers are obliged to report to the court. Our research tools looked into health, social background and a range of psychosocial competencies determining good care of the child in kinship care. Any inadequacies revealed in this respect would provide grounds for the court to dissolve the foster relationship. Even though the interviewer provided extensive explanation about research confidentiality, this factor had a significant effect on raising unjustified concerns in some foster families that the survey might make them “look bad” and result in them losing the foster care of their grandchildren. Another limitation was the relatively long data collection process due to the large size of the research tools used and the respondents’ age. Domestic and care-related responsibilities, fatigue and disease burden meant that sometimes it was necessary to meet with the foster family on several different occasions.

## Data Availability

The datasets used and/or analysed during the current study are available from the first author on reasonable request.
